# Toxoplasmosis-Associated Hemophagocytic Lymphohistiocytosis in a Liver Transplant Recipient

**DOI:** 10.7759/cureus.71843

**Published:** 2024-10-19

**Authors:** Yu Gong, Ting Wang

**Affiliations:** 1 Department of Critical Care Medicine, Zhongshan Hospital, Fudan University, Shanghai, CHN; 2 Department of Clinical Care Medicine, Zhongshan Hospital, Fudan University, Shanghai, CHN

**Keywords:** diagnosis & prognosis, hemophagocytic lymphohistiocytosis, liver transplant, metagenomic next-generation sequencing, toxoplasmosis

## Abstract

Toxoplasmosis is a rare parasitic infectious disease in solid organ transplant recipients. The disease is characterized by difficulties in diagnosis and high mortality. However, there have been no reported cases of hemophagocytic lymphohistiocytosis (HLH) caused by toxoplasmosis in liver transplant patients. Here, we present the case of an adult female liver transplant recipient who experienced a fatal outcome and developed secondary HLH following confirmation of toxoplasmosis through metagenomic next-generation sequencing. The patient exhibited symptoms, including high fever, skin rash, unconsciousness, and multiorgan failure. The condition met six out of eight criteria for HLH according to the HLH-2004 diagnostic criteria. Additionally, the H-score for this patient was 287 points, confirming the diagnosis of HLH. This represents the first reported case of toxoplasmosis-associated secondary HLH in an adult liver transplant recipient in China.

## Introduction

Compared to heart and kidney transplants, the incidence of liver transplants associated with toxoplasmosis is relatively rare. A study found that 8 out of 13 liver transplant patients with toxoplasmosis died [1,2]. The infection sources [3] included donor-derived infection, reactivation of latent infection, or primary infection. In immunosuppressed recipients with toxoplasmosis, the triggering of secondary hemophagocytic lymphohistiocytosis (HLH) may lead to fatal outcomes [4], even with accurate diagnosis and effective treatments. According to previous reports [5] of immunosuppressed recipients with toxoplasmosis, high fever and neurological syndromes may suggest the existence of toxoplasmosis, but confirmation requires evidence of the parasite and antibodies. Due to non-specific symptoms in immunosuppressed recipients with toxoplasmosis with HLH, an early diagnosis is crucial.

## Case presentation

Upon admission on November 27th, 2021, a 42-year-old Chinese female patient with hepatitis B was diagnosed with hepatitis B virus-related acute-on-chronic liver failure and hepatic encephalopathy. The serological test revealed abnormal results, and the patient’s Model for End-Stage Liver Disease was 28 points. Subsequently, on December 5th, 2021, the patient underwent orthotopic liver transplantation and received an immunosuppression regimen consisting of tacrolimus, mycophenolate mofetil, and corticosteroids with a target trough concentration of tacrolimus ranging from 3 to 5 ng/mL.

Her recovery was unfortunately eventful, with fluctuations in total bilirubin levels ranging between 100 and 300 μmol/L accompanied by fever and oliguria. Abdominal drainage fluid and blood cultures indicated the presence of multidrug-resistant *Klebsiella pneumoniae*, *Enterococcus faecalis*, and *Candida parapsilosis*. Additionally, cytomegalovirus (CMV) was detected at levels ranging from 1.9 × 10^3^ to 4.01 × 10^4^ copies/mL. After receiving antimicrobial therapy including ceftazidime-avibactam, caspofungin for fungal infection, and ganciclovir for antiviral treatment, she had her body temperature return to normal, and her vital signs stabilized; however, she continued experiencing oliguria requiring daily renal replacement therapy.

From January 9th, 2022 (postoperative day (POD)34) onwards, the patient exhibited mental changes such as delirium and reticence despite normal cerebral CT scans. Her first cerebral MRI on January 14th, 2022 (POD39) showed a couple of lacunar ischemic lesions in the bilateral frontal lobe and a few microhemorrhages in the left frontal lobe. By January 27th, 2022 (POD52), there was no improvement in her mental state. Her condition suddenly deteriorated on February 8th, 2022 (POD63), manifesting as high fever (40℃), low blood pressure, and transient unconsciousness. Drainage fluid samples of bile and blood were obtained for metagenomic next-generation sequencing (mNGS) analysis again. A second cerebral MRI revealed thalamic lesions that may have involved the brain stem. Four days later, she fell into a coma and required endotracheal intubation; however, her vital signs worsened rapidly, and the serological test results declined (Table 1).

**Table 1:** The changing patterns of diverse biochemical parameters in the patient suspected of having HLH. WBC: white blood cell; TB: total bilirubin; AST: aspartate aminotransferase; PT: prothrombin time; POD: postoperative day; HLH: hemophagocytic lymphohistiocytosis

	2.8 (POD63)	2/9 (POD64)	2/10 (POD65)	2/11 (POD66)	2/12 (POD67)	2/13 (POD68)	2/14 (POD69)	2/15 (POD70)	2/16 (POD71)	2/17 (P0D72)
Hemoglobin (g/L)	71	59	93	60	41	48	48	54	51	38
Platelet (×10^9^/L)	128	100	78	41	20	27	29	9	64	20
WBC (×10^9^/L)	3.5	1.97	4.37	3.47	8.22	10.08	7.25	2.92	6.55	4.55
TB (μmol/L)	56.7	67.9	81.9	91.9	157.2	187.5	190.0	215.3	199.1	190.8
AST (U/L)	57	109	210	409	>7,500	6,853	3,325	1,967	1,250	887
PT (s)	22.2	29.6	51.7	>170	>170	42.5	>170	>170	69.2	>170
Procalcitonin (ng/mL)	-	2.98	7.01	3.95	0.9	0.56	0.46	0.27	0.27	0.13

**Figure 1:**
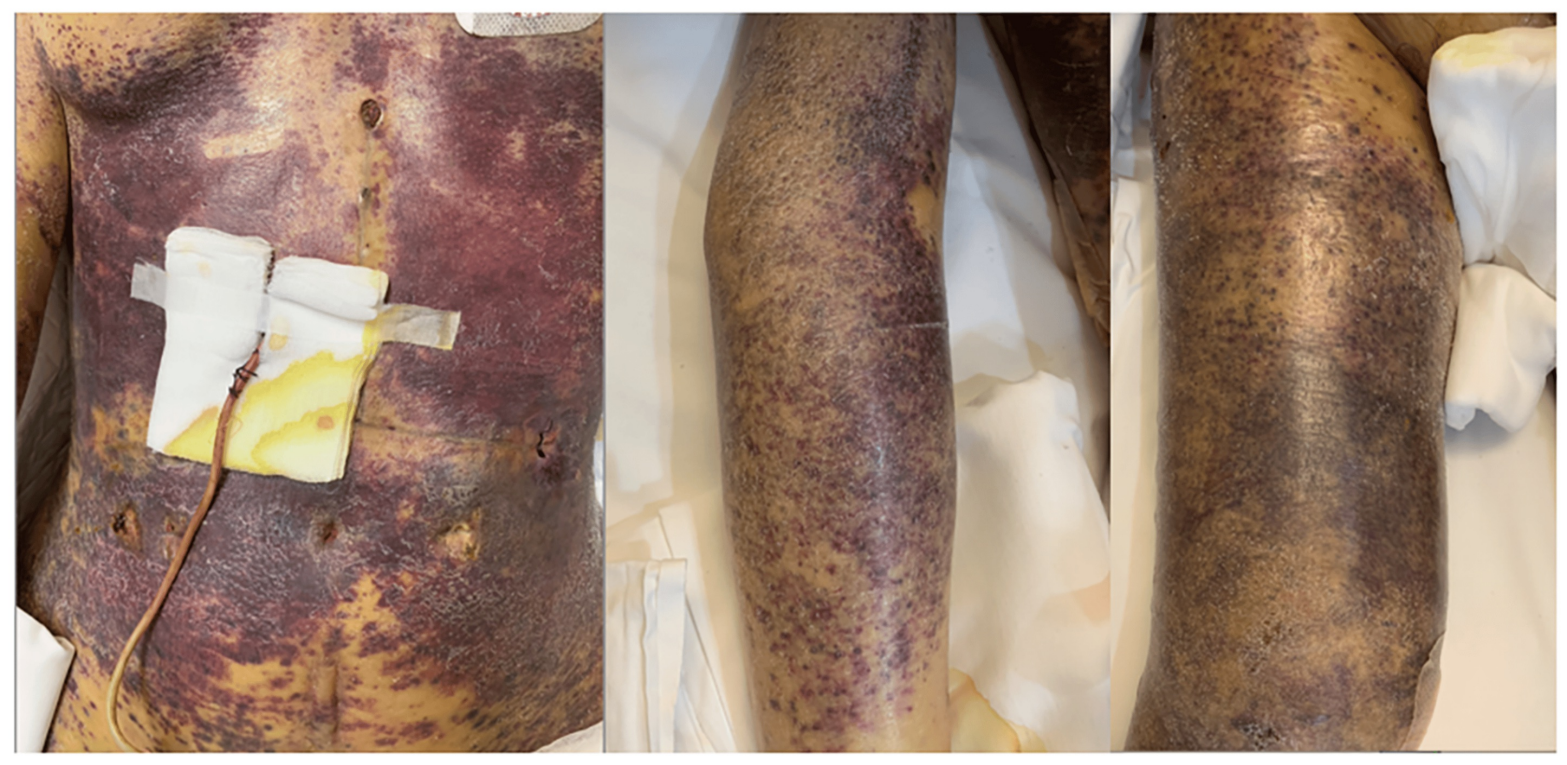
After being readmitted to the intensive care unit, the patient developed extensive ecchymosis on the trunk and extremities.

Furthermore, extensive ecchymosis was observed on the patient’s skin, involving the chest, abdomen, and limbs (Figure 1). Considering the sharp decrease in leukocyte, platelet, and erythrocyte counts accompanied by high fever, unconsciousness, and multiple organ dysfunction, the possibility of secondary HLH could not be excluded. According to the HLH-2004 diagnostic criteria, six out of eight criteria were met, namely, triglyceride levels at 8 μmol/L, ferritin >2,000 ng/mL, high fever, interleukin-2 receptor >7,500 U/L, fibrinogen <40 mg/mL, and decreased leukocyte count along with platelet and erythrocyte counts. The H-score for the patient was 287 points, confirming the diagnosis of HLH. The patient underwent plasma exchange therapy, methylprednisolone treatment, and oXiris therapy. Due to multiple organ failure, she was not eligible for standard HLH treatments involving dexamethasone and etoposide. Unexpectedly, her blood sample revealed abundant *Toxoplasma gondii* sequences through mNGS, despite her negative serum toxoplasma IgM but positive IgG. Unfortunately, despite adding sulfamethoxazole and azithromycin, her vital signs rapidly deteriorated, leading to her death on February 17th, 2022 (POD72).

## Discussion

The challenges posed by latent infection and non-specific clinical symptoms complicate the diagnosis of toxoplasmosis in liver transplant patients [6], especially when accompanied by secondary HLH. The link between liver transplantation and toxoplasmosis was first reported by Antony in 1972. *Toxoplasma gondii* transmission during liver transplants can occur through donor transmission, reactivation of latent infection, or primary infection, resulting in various clinical manifestations such as pneumonia, meningitis, and multisystem organ failure. Diagnosis usually involves detecting specific IgM or identifying the parasite histologically from pathological tissue samples. However, serological tests may not be reliable for immunosuppressed patients due to potential false-negative results; therefore, postmortem confirmation is often necessary. Currently, mNGS [7-9] is widely utilized and holds significant importance in identifying infectious sources. There should be a heightened focus on assessing parasite antibodies in both liver transplant donors and recipients [10]. The debate about whether prophylactic sulfamethoxazole therapy should be strongly recommended for liver transplant recipients without contraindications remains unresolved.

Overall, 80% of primary HLH patients manifest their first symptoms before the age of two, encompassing a series of clinical manifestations initially documented in 1939 by Scott and Robb-Smith [11]. Conversely, secondary HLH is more common in adult patients who often have underlying etiologies such as autoimmune diseases, infections, malignant tumors, or immunosuppression; however, recognizing and diagnosing the disease poses great challenges. We need to consider diagnosing secondary HLH when immunosuppressed patients’ laboratory tests and vital signs deteriorate rapidly due to severe infection [12]. Until 2022, a total of 20 cases of adult liver transplant with secondary HLH were reported [13,14] with mortality rates reaching as high as 85% (17/20). Triggers include viral, fungal, and tuberculosis infections [15]; small liver syndrome; and autoimmune diseases. A successful clinical case involving the use of ruxolitinib in an adult liver transplant patient with secondary HLH following a CMV infection was reported in 2022 when standard HLH therapy proved ineffective and resulted in deteriorating liver function [12,13]. It is important to emphasize that the dosage of immunosuppressive drugs should be appropriately reduced for high-risk patients.

## Conclusions

Our case report emphasizes the critical need for specific surveillance and prophylaxis of toxoplasmosis in liver transplant recipients. We recommend using trimethoprim/sulfamethoxazole prophylaxis during the early post-transplantation period. Additionally, it is important to recognize that HLH, though rare, can complicate toxoplasmosis and may be prone to misdiagnosis. Both the prevention of secondary HLH and the development of more effective therapeutic drugs are key objectives for clinical research. Toxoplasmosis in liver transplant patients may present diagnostic challenges due to a lack of definitive evidence for parasite infection and its potential association with various underlying diseases. Suspicion should also be raised when unexplained fever and/or neurological syndromes occur. We recommend bone marrow aspiration, cerebral MRI, serological examinations for toxoplasma antigens, and mNGS as valuable tools for diagnosing toxoplasmosis-induced HLH in immunosuppressed patients.
